# KRT14 marks a subpopulation of bladder basal cells with pivotal role in regeneration and tumorigenesis

**DOI:** 10.1038/ncomms11914

**Published:** 2016-06-20

**Authors:** George Papafotiou, Varvara Paraskevopoulou, Eleni Vasilaki, Zoi Kanaki, Nikolaos Paschalidis, Apostolos Klinakis

**Affiliations:** 1 Center for Basic Research, Biomedical Research Foundation of the Academy of Athens, Athens, Greece; 2 Laboratory of Cellular Immunology, Center for Basic Research, Biomedical Research Foundation of the Academy of Athens, Athens, Greece

## Abstract

The urothelium is a specialized epithelium that lines the urinary tract. It consists of three different cell types, namely, basal, intermediate and superficial cells arranged in relatively distinct cell layers. Normally, quiescent, it regenerates fast upon injury, but the regeneration process is not fully understood. Although several reports have indicated the existence of progenitors, their identity and exact topology, as well as their role in key processes such as tissue regeneration and carcinogenesis have not been clarified. Here we show that a minor subpopulation of basal cells, characterized by the expression of keratin 14, possesses self-renewal capacity and also gives rise to all cell types of the urothelium during natural and injury-induced regeneration. Moreover, these cells represent cells of origin of urothelial cancer. Our findings support the hypothesis of basally located progenitors with profound roles in urothelial homoeostasis.

## Abstract (web summary)

It is unclear whether there is a progenitor/stem cell in the basal layer of the urothelium in the bladder. Here, the authors identify Keratin14 positive cells that can regenerate the bladder in both a natural and injury-induced manner, and following neoplastic transformation, can give rise to tumours.

## Graphical abstract

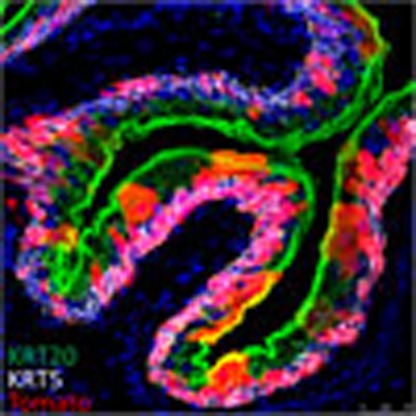

The urothelium is a slowly cycling tissue consisting of basal, intermediate and superficial or umbrella cells that form the urine-blood barrier^1^. Tissue regeneration following microbial or chemical injury relies upon proliferation of progenitor cells^2^,^3^. Whether the repair process is mediated by a single basal progenitor co-expressing sonic hedgehog (SHH) and keratin 5 (KRT5)^4^, or by distinct basal and intermediate progenitors that regenerate the basal and umbrella layers, respectively^5^,^6^, without lineage crossing, has become a controversial issue.

In humans, cells expressing KRT14 (keratin 14; KRT14^pos^) are considered the most primitive population in bladder cancer^7^,^8^, and are enriched upon consecutive rounds of chemotherapy^9^. In a mouse model of invasive bladder cancer, KRT14^pos^ cells are preferentially amplified upon STAT3 overexpression^10^. Nevertheless, KRT14^pos^ cells are not yet described in normal human urothelium, while definitive proof that KRT14^pos^ cells correspond to urothelial progenitors in mice remains elusive. Moreover, potential roles of these cells in tissue homoeostasis and regeneration are yet to be investigated.

Here we provide unequivocal evidence that a small subset of basal cells of embryonic origin characterized by KRT14 expression are the stem cells of the bladder. Using *in vivo* lineage-tracing experiments in mice, and *in vitro* clonogenic and explant cultures, we show that KRT14^pos^ cells participate both in natural and injury-induced bladder regeneration by giving rise to all layers. Finally, upon neoplastic transformation, KRT14^pos^ cells give rise to a spectrum of tumours, implicating them as the cells of origin of bladder cancer. These findings will inspire future studies regarding their role in normal bladder homoeostasis and disease, and their use in regenerative medicine applications.

## Results

### KRT14 marks a dynamic basal urothelial subpopulation

**Figure 1:**
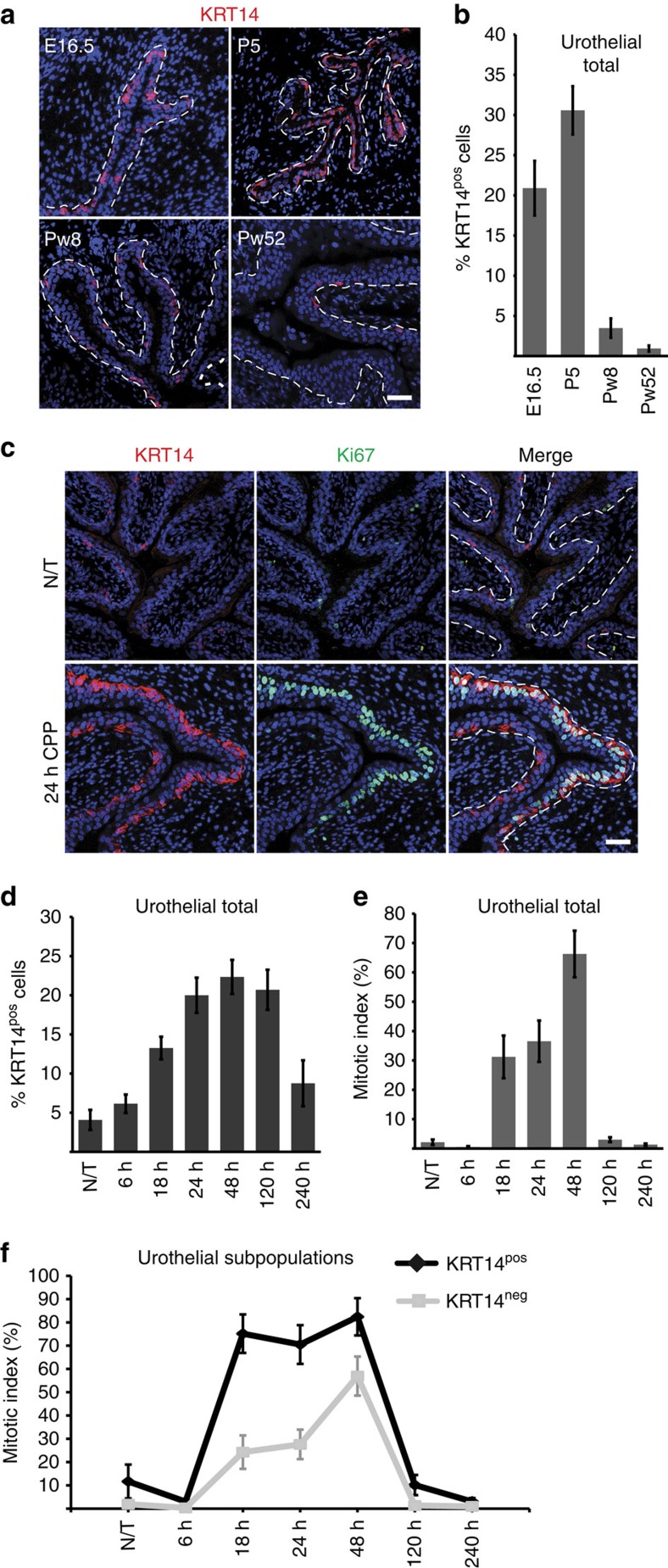
Dynamic changes in the number of KRT14^pos^ cells in the normal development and tissue repair upon injury. (**a**) Immunofluorescence (IF) against KRT14 on bladders from E16.5, P5, Pw8 and Pw52 bladders. (**b**) Quantification of KRT14^pos^ cells at various developmental stages. Percentages are averages from three mice, and the total cells counted were 1,426, 1,669, 2,213 and 1,703 for E16.5, P5, Pw8 and Pw52, respectively. (**c**) IF against KRT14 and Ki67 on the bladder from an 8-week-old mouse before, and at 24 h after CPP injection. (**d**) Quantification of KRT14^pos^ cells at 6, 18, 24, 48, 120 and 240 h post CPP injection. (**e**) Quantification of urothelial cell proliferation at the above time points post CPP injection. (**f**) Quantification of proliferation of KRT14^pos^ and KRT14^neg^ urothelial cells during CPP-induced injury and repair. Numbers in **b**, **d** and **e** are expressed as mean of percentages over total urothelial cells±s.e.m. Numbers in **f** are mean of percentages over KRT14^pos^ or KRT14^neg^ cells±s.e.m. For **b**, **d**, **e** and **f**, two group comparison was performed using the Student's *t*-test and the number of samples and *P* values can be found in the text, in Table 1 and in Supplementary Tables 1 and 2, respectively. For **b**, **d** and **e**, multiple comparison using Kruskal–Wallis test was also performed and *P* values were 0.0007, <0.0001 and 0.0001, respectively. Dash lines represent the basement membrane. Scale bars, 50 μm. N/T, not treated.

In the adult mouse urothelium, KRT5 expression marks basal cells that constitute ∼90% of all urothelial cells, while terminally differentiated umbrella cells are marked by the expression of keratin 20 (KRT20)^11^,^12^. KRT14 protein is observed for the first time on E16.5 embryos in a subset (20.89±3.4%) of strictly basal cells (Fig. 1a,b) that also express KRT5 (ref. 5; Supplementary Fig. 1a). KRT14^pos^ cells remain exclusively basal throughout life, while their numbers peak postnatally, amounting to 30.6±3% of total, and decrease steadily during adulthood to 3.5±1.2% (*P*<0.01) at 8 weeks and 0.9±0.5% (*P*<0.001) at 1 year of age (Fig. 1b).

Within 6 h of chemical injury with cyclophosphamide (CPP)^2^, damage and exfoliation of KRT20^pos^ cells occurs (Supplementary Fig. 1b), to be followed by a marked increase of KRT14^pos^ cell numbers, peaking at 48 h post CPP injection to 22.3±2.2% and declining soon after tissue repair (Table 1; Fig. 1c,d; Supplementary Fig. 1c). Ki67 staining indicates that cell proliferation commences ∼18–24 h post CPP injection and is initially restricted to the basal layer (Fig. 1c,e; Supplementary Fig. 1c; Supplementary Table 1). Interestingly, between 18 and 24 h, when the umbrella cell layer is largely absent, the mitotic index of KRT14^pos^ cells is approximately threefold higher than that of KRT14^neg^ cells. As proliferation seems to be spreading to non-basal cells by 48 h, this difference drops to a still statistically significant 1.4-fold (Fig. 1f; Supplementary Fig. 1c; Supplementary Table 2).

### Genetic labelling and lineage tracing of KRT14^pos^ cells

**Figure 2:**
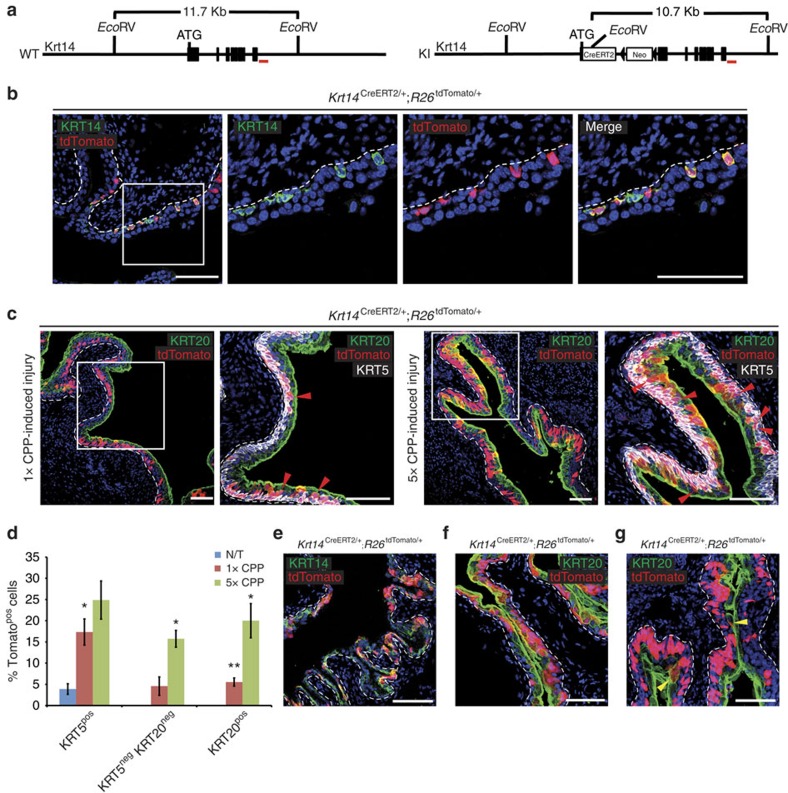
KRT14^pos^ cells give rise to all urothelial lineages during both normal tissue turnover and chemical injury. (**a**) Targeting strategy of the *Krt14* locus with a CreERT2/Neo cassette. (**b**) Permanent labelling of KRT14^+^ basal urothelial cells by activation of a tdTomato transgene, by injecting an 8-week-old *Krt14*^CreERT2/+;^*R26*^tdTomato/+^ mouse with tamoxifen for five consecutive days and sacrificing 2 days later. (**c**) Sections of bladders of *Krt14*^CreERT2/+;^*R26*^tdTomato/+^ mice subjected to either one or five rounds of CPP-induced injury and full recovery. Red arrowheads point to Tomato^pos^/KRT5^neg/low^/KRT20^neg^ intermediate cells. (**d**) Graph showing percentages of Tomato^pos^ basal (KRT5^pos^), intermediate (KRT5^neg^KRT20^neg^) and luminal (KRT20^pos^) cells in bladders from tamoxifen-injected, non CPP-treated *Krt14*^CreERT2/+;^*R26*^tdTomato/+^ mice or from *Krt14*^CreERT2/+;^*R26*^tdTomato/+^ mice after one or five rounds of CPP-induced injury and full recovery. Numbers are mean of percentages±s.e.m. Two group comparison was performed using the Student's *t*-test and the number of samples and *P* values can be found in Table 2. *P<0.05; **P<0.01. Multiple comparison using Kruskal–Wallis test was also performed and *P* values were 0.0107, 0.0014 and 0.0005 for Tomato^pos^ basal (KRT5^pos^), intermediate (KRT5^neg^KRT20^neg^) and luminal (KRT20^pos^) cell populations, respectively. (**e**) Sections of *Krt14*^CreERT2/+;^*R26*^tdTomato/+^ bladders from mice treated with tamoxifen at E16.5, and subsequently killed at either P5 without injury, or (**f**) after a round of CPP-induced injury and full recovery at Pw8. (**g**) Section of a bladder from a *KRT14*^CreERT2/+;^*R26*^tdTomato/+^ mouse killed at 10 months of age without injury. With the exception of embryonic labelling (**e**,**f**) the rest of the mice were injected with tamoxifen at the age of 8 weeks. Dash lines represent the basement membrane. Scale bars, 100 μm. N/T, not treated.

To perform lineage-tracing experiments *in vivo*, we generated a knock-in CreERT2 recombinase line into the *Krt14* locus (Fig. 2a). CreERT2 insertion disrupts the open reading frame of the locus leading to a null allele. Tamoxifen administration in *Krt14*^CreERT2/+^;*R26*^tdTomato/+^ bitransgenic mice identifies a subset of basal cells that co-express KRT14 and Tomato indicating faithful CreERT2 expression (Fig. 2b). As indicated by the existence of KRT14^pos^ Tomato^neg^ cells, the *R26*^tdTomato^ allele is not recombined in all KRT14^pos^ cells. The most obvious explanation for this discrepancy is that either tamoxifen local concentration or Krt14 expression levels fail to reach an effective threshold. Vehicle-treated control mice fail to produce Tomato-positive cells (Supplementary Fig. 2), indicating a tightly regulated Cre recombinase driver.

### KRT14^pos^ cells give rise to all urothelial lineages

Tamoxifen administration followed by a single CPP injection and recovery of *Krt14*^CreERT2/+^;*R26*^tdTomato/+^ bitransgenic mice shows a significant increase of Tomato^pos^ cells in the basal layer, and for the first time in the umbrella layer (Fig. 2c,d; Table 2). Of note, after a single injection with CPP, the immediate descendants of KRT14^pos^ cells initially remain basal/intermediate, as indicated by the sharp increase in Tomato^pos^/KRT5^pos^ frequency (from 3.89±1.25% to 17.33±3.07%) and their relative absence from the umbrella layer (Fig. 2c,d; Table 2). Given the fact that upon CPP treatment, all umbrella cells need to be replenished, the scarcity of Tomato^pos^/KRT20^pos^ cells (Fig. 2d; Table 2) implies the existence of a non KRT14 cell population that initially mediates umbrella layer regeneration. Upon repeated cycles (5 ×) of CPP injection and recovery; however, Tomato^pos^ cells become quite abundant in all cell layers (Fig. 2c,d; Table 2), indicating that KRT14^pos^ cells are primitive cells that outlast and can give rise to all other cell types.

*In utero* labelling followed by chase through adulthood reveals that postnatal (P5) KRT14^pos^ cells are derived directly from their embryonic counterparts (Fig. 2e). Moreover, Tomato^pos^ descendants of embryonically labelled KRT14^pos^ cells repopulate CPP-injured bladders and give rise to all cell types (Fig. 2f). Eight-month long chase experiments in *Krt14*^CreERT2/+^;*R26*^tdTomato/+^ bitransgenic mice injected with tamoxifen at the age of 8 weeks indicate that KRT14^pos^ cells participate in the natural regeneration of all urothelial layers (Fig. 2g). Altogether, our data indicate that Krt14 expression marks an embryonic subpopulation of cells that persists through adulthood and participates both in natural cycling, and repair upon injury. The KRT14^pos^ subpopulation gives rise to all cell types in the mouse urinary bladder.

### KRT5^pos^ basal cells regenerate the umbrella layer

**Figure 3:**
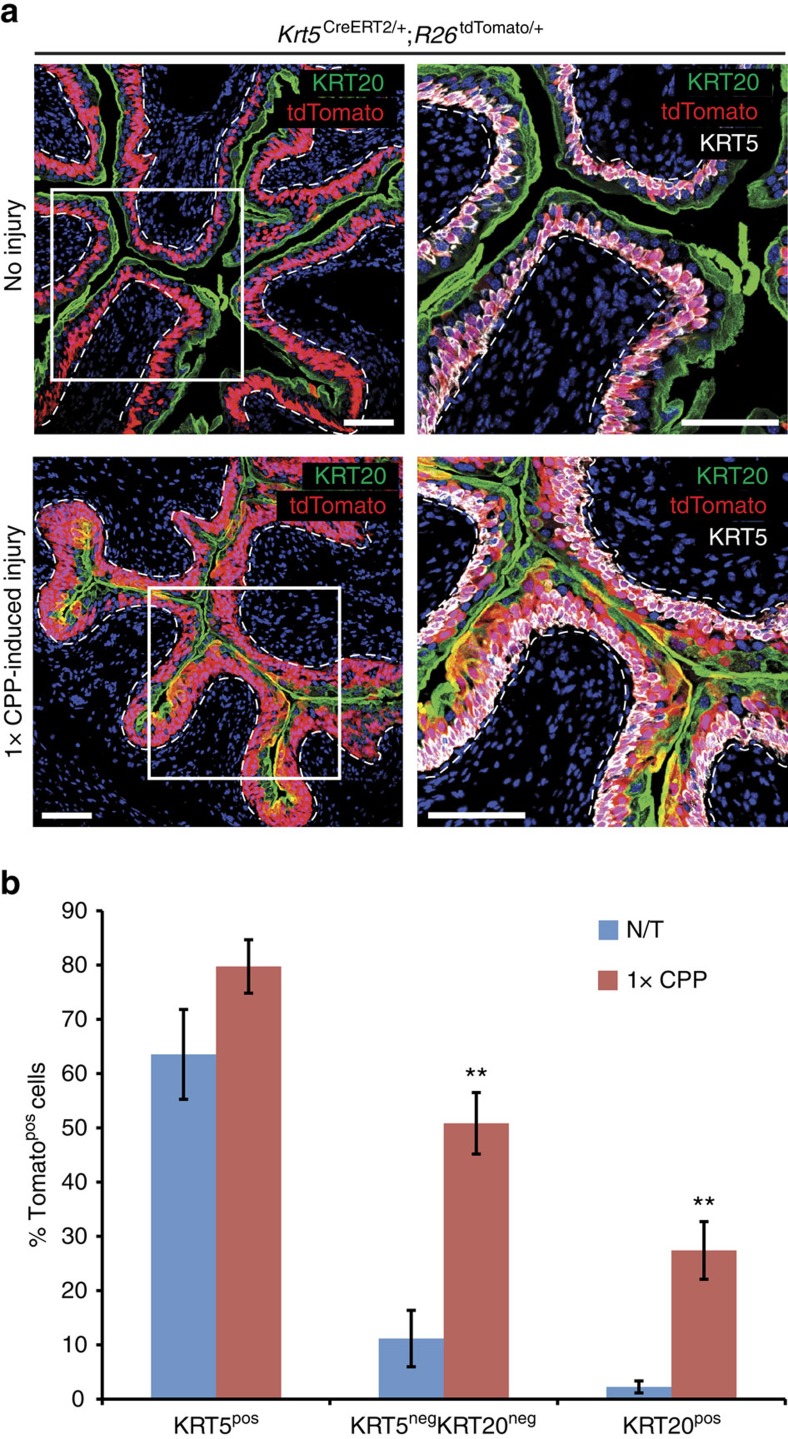
KRT5^pos^ cells give rise to umbrella cells following CPP injury. (**a**) Sections of bladders from Krt5^CreERT2/+^;R26^tdTomato/+^ mice with or without subsequent single round of CPP-induced injury and full recovery. Mice were injected with tamoxifen at the age of 8 weeks. Dash lines represent the basement membrane. Scale bars, 100 μm. (**b**) Graph showing percentages of Tomato^pos^ basal (KRT5^pos^), intermediate (KRT5^neg^KRT20^neg^) and luminal (KRT20^pos^) cells in bladders from tamoxifen-treated Krt5^CreERT2/+^;R26^tdTomato/+^ mice, either non CPP-treated or after a single round of CPP-induced injury and full recovery. Numbers are mean of percentages±s.e.m. For **b**, two group comparison was performed using the Student's *t*-test and the number of samples and *P* values can be found in Supplementary Table 3. **P<0.01. N/T, not treated.

Our observation that KRT14^pos^ basal cells participate in umbrella layer repair is in agreement with findings, showing that SHH^pos^ cells in the basal layer give rise to umbrella cells upon chemical and uropathogenic bacteria-induced damage^4^. It contradicts, however, a previous report showing that KRT5^pos^ cells, which encompass the KRT14^pos^ subpopulation, do not contribute to the umbrella layer regeneration^5^. To clarify this issue, we performed lineage-tracing experiments using a *Krt5*^CreERT2^ transgenic mouse line and found that KRT5^pos^ cells contribute to umbrella layer regeneration following a single challenge with CPP (Fig. 3a,b; Supplementary Table 3). Given the fact that both studies have used the same *Krt5*^CreERT2^ driver^13^, we hypothesize that the *R26*^tdTomato^ reporter used in this study is more prone to recombination than the *R26*^Tomato/gfp^ used in the Gandhi *et al* study. In support of this hypothesis, variable labelling efficiency has been reported with the *Krt5*^CreERT2^ driver (60 and 39% in two different studies^5^,^14^ with *Krt5*^CreERT2^;*R26*^Tomato/gfp^ mice, and an even lower 29% with *Krt5*^CreERT2^;*R26*^LacZ^ mice^14^), while we observe a 63.5% labelling efficiency (all percentages are calculated as the fraction of reporter-positive cells that immunostain with anti-KRT5 antibodies). The existence of a small subpopulation of KRT5^pos^ cells, which, however, express lower levels of Krt5 (KRT5^low^)^15^ and therefore label more poorly, is a plausible explanation for this difference. In support of this hypothesis, immunofluorescence experiments identify a small population of KRT5^low/neg^/KRT20^neg^ Tomato-labelled intermediate cells that could correspond to the ‘I' cells that were previously described as umbrella layer progenitors^5^ (Fig. 2c).

### KRT14^pos^ cells support *ex vivo* growth of bladder explants

**Figure 4:**
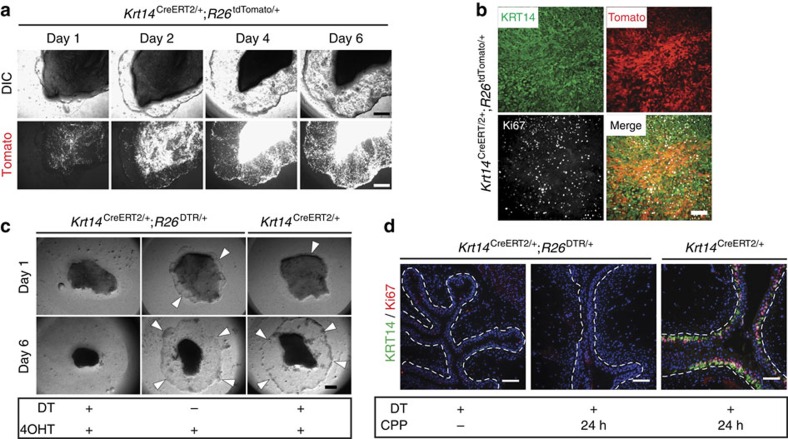
KRT14^pos^ cells support growth in *ex vivo* bladder tissue explant cultures. (**a**) Urothelial explant cultures of a tamoxifen-injected *Krt14*^CreERT2/+^;*R26*^tdTomato/+^ 8-week-old mouse at 1, 2, 4 and 6 days. The tissue was obtained and cultured 2 days after the last tamoxifen injection. (**b**) KRT14 and Ki67 staining of the 6-day outgrowth of the explant shown in **a**. (**c**) Urothelial explant cultures of tissues obtained from tamoxifen-injected 8-week-old *Krt14*^CreERT2/+^;*R26*^DTR/+^ or *Krt14*^CreERT2/+^ mice, cultured either in the presence or absence of diphtheria toxin (DT). Four-hydroxytamoxifen (4OHT) was included in the culture medium for the first 12 h to ensure complete transgene recombination. (**d**) IF against KRT14 and Ki67 on bladders of experimental (*Krt14*^CreERT2/+^;*R26*^tdTomato/+^) and control (*Krt14*^CreERT2/+^) mice challenged with CPP and injected with DT. Scale bars, 1 mm (**a**,**c**); 100 μm (**b**,**d**).

To assess the proliferative potential of KRT14^pos^ cells and their contribution to tissue growth, we employed an *ex vivo* assay using bladder tissue explants^16^. When seeded onto polyester filters, these explants produce outgrowths spreading and covering the filter surface within days. We dissected and grew explants from *Krt14*^CreERT2/+^;*R26*^tdTomato/+^ mice injected with tamoxifen before sacrifice. We observed a massive expansion of the Tomato-labelled population, both on the explanted tissue and the newly formed outgrowth (Fig. 4a). Ki67 and KRT14 immunostaining showed that outgrowth cells are actively proliferating and are nearly all KRT14^pos^, as well as KRT5^pos^ (Fig. 4b; Supplementary Fig. 3a). Extensive Tomato labelling of KRT14^pos^ outgrowths (Fig. 4a,b) indicates that this population represents the lineage of *in vivo*-labelled KRT14^pos^ basal cells, rather than a population with newly acquired Krt14 expression. This is supported by the fact that bladder explants from *Krt14*^CreERT2/+^;*R26*^tdTomato/+^ mice, which were treated with vehicle, present extremely rare Tomato-positive cells corresponding to ‘leaky' recombination, while explants from the same bladder treated with 4-hydroxytamoxifen (4OHT) *in vitro* produce explants with extensive Tomato fluorescence (Supplementary Fig. 3b).

Conditional ablation of KRT14^pos^ cells in tissues explanted from *Krt14*^CreERT2/+^;*R26*^DTR/+^ mice^17^ and treated with diphtheria toxin (DT), completely prevented explant growth, while explants from these mice grew indistinguishably from their wild-type counterparts (*Krt14*^CreERT2/+^ or *R26*^DTR/+^) in the absence of DT (Fig. 4c).

### KRT14^pos^ cells originate from themselves during injury

To trace the origin of KRT14^pos^ cells *in vivo* during regeneration, *Krt14*^CreERT2/+^;*R26*^DTR/+^ mice were challenged with CPP upon DT-mediated ablation of KRT14^pos^ cells. Despite the obvious tissue damage, no proliferation was observed, while the KRT14 population was practically extinct (Fig. 4d). This implies that the KRT14 cell pool is regenerated exclusively from KRT14^pos^ cells. Unfortunately, premature mouse dying due to fatal complications in other tissues expressing KRT14 prevented us from monitoring mice through a complete round of injury and recovery, and thus to assess the effect of KRT14^pos^ cell absence in bladder regeneration.

### Clonogenic and differentiation capacity of KRT14^pos^ cells

**Figure 5:**
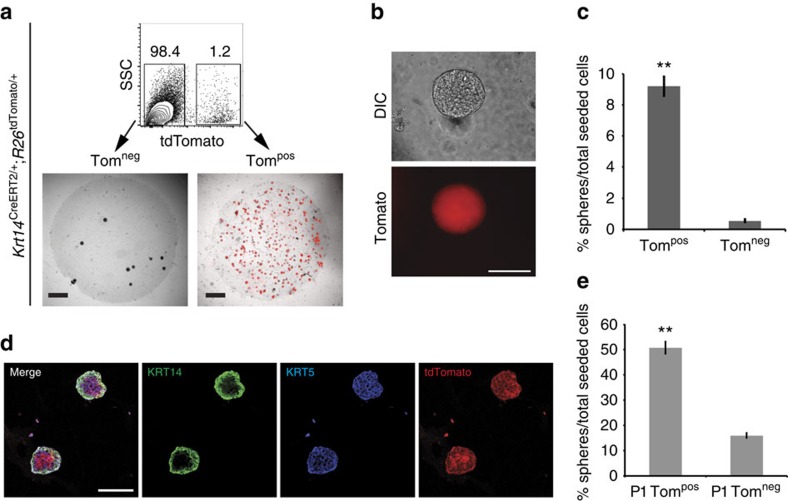
KRT14^pos^ cells show increased *in vitro* clonogenic capacity. (**a)** Tomato^neg^ (Tom^neg^) and Tomato^pos^ (Tom^pos^) cells from tamoxifen-injected 8-week-old mice, were FACS sorted with Tomato positivity using a Becton Dickinson FACS Aria IIu cell sorter, and cultured in Matrigel. Cultures at 11 days are shown. (**b**) High magnification of a Tom^pos^ sphere from **a**. (**c**) Sphere-forming efficiency of Tom^pos^ (9.21±0.61%, *n*=10) and Tom^neg^ (0.56±0.09%, *n*=10) urothelial cells from **a**. (**d**) IF against KRT5 and KRT14 of Matrigel-grown Tom^pos^ spheres indicating stratification. (**e**) Sphere forming efficiency of passaged (P1) Tom^pos^ (50.88±2.37%, *n*=10) and Tom^neg^ (16.04±0.94%, *n*=9) urothelial cells obtained from Matrigel cultures from **a**. For **c** and **e**, two group comparison was performed using the Student's *t*-test. **P<0.0001. Scale bars represent 1 mm in **a**, and 100 μm in **b** and **d**.

To measure the clonogenic capacity of KRT14^pos^ cells *in vitro*, we generated bladder single cell suspensions from *Krt14*^CreERT2/+^;*R26*^tdTomato/+^ mice that were injected with tamoxifen before tissue digestion. Fluorescent-activated cell sorting (FACS) indicated that Tomato^pos^ cells represent 1.2% of the total population (Fig. 5a). When seeded on Matrigel in clonogenic densities, these cells produce perfect spheres within 2 weeks (Fig. 5a,b). Fluorescence microscopy revealed that the sphere-forming capacity of KRT14^pos^ (Tom^pos^) cells is significantly higher (*P*<0.0001) than KRT14^neg^ (Tom^neg^) cells (9.21±0.61% versus 0.56±0.09%; Fig. 5c). After 4 weeks in culture, expression of KRT14 is restricted in the outer layer of spheres, while KRT5 and Tomato are expressed throughout (Fig. 5d). This illustrates that *in vitro,* KRT5^pos^KRT14^neg^ cells differentiate from KRT14^pos^ cells, and this stratification is reminiscent of what is observed *in vivo*. Passaging of both populations and reculturing on Matrigel showed that KRT14^pos^ cells retain their *in vitro* proliferative capacity (Fig. 5e). These data indicate that the clonogenic capacity of urothelial cells reside by large within the KRT14 compartment. Combined, our data indicate that KRT14^pos^ cells give rise to themselves and other cell types both *in vivo* and *in vitro*.

### Wnt/β-catenin signals support KRT14^pos^ cell proliferation

**Figure 6:**
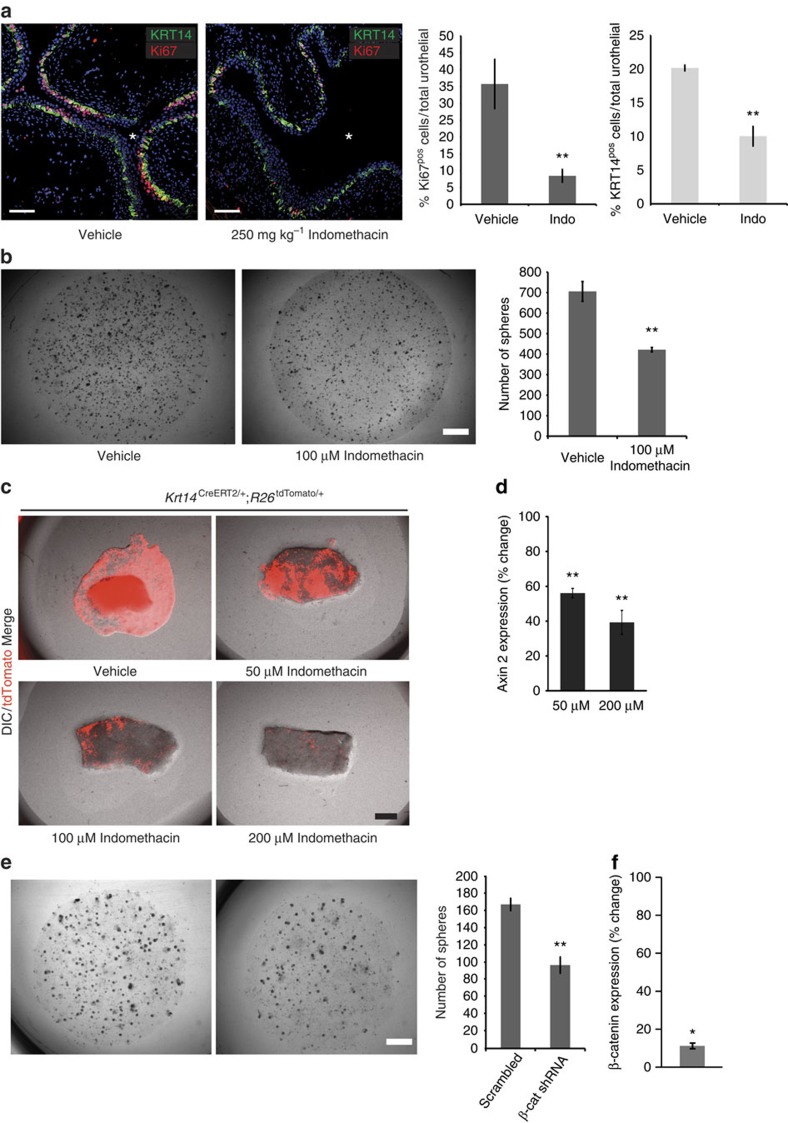
Active Wnt/β-catenin signaling pathway is necessary for KRT14^pos^ cell proliferation and bladder repair upon injury. (**a**) *In vivo* treatment with 250 mg kg^−1^ indomethacin reduces CPP-induced proliferation (8.47±2.05% from 35.74±7.3% in vehicle-treated controls) and KRT14^pos^ cell numbers (24 h after injury; 10.04±1.45% from 20.17±0.43% in vehicle-treated controls). Data are means of percentages ±s.e.m from four vehicle, or five Indomethacin-treated mice. Total cells counted were 5,108 and 9,947, respectively. (**b**) Clonogenic assays on Matrigel of total bladder populations cultured in the presence (421.6±10.8) or absence (705±48.4) of 100 μM indomethacin. Data are averages of five independent cultures. (**c**) Bladder explant cultures with increasing concentrations of indomethacin. (**d**) Axin 2 transcript levels (quantitative PCR data) in 3- day explants treated with 50 (56.1±2.7%) and 200 μM indomethacin (39.3±6.9%) plotted as percentage of Axin2 levels from vehicle-treated explants. (**e**) Clonogenic assays on Matrigel of bladder populations (10,000 cells) stably transduced with lentiviral vectors expressing a scrambled (167.38±7.14, *n*=8) or an anti-β-catenin (β-cat) shRNA (96.86±9.49, *n*=7). (**f**) Expression levels of β-catenin in 2-week total Matrigel cultures of primary bladder cells expressing anti-β-catenin (β-cat) shRNA (10.86±1.48%) plotted as percentage of β-cat levels from Matrigel cultures of primary bladder cells expressing scrambled shRNA. All data are mean values±s.e.m. For **a**, **b** and **e**, two group comparison was performed using the Student's *t*-test and *P<0.05; **P<0.01. Stars designate the bladder lumen.

Previous reports have implicated the Wnt/β-catenin signalling pathway in regulating basal cell proliferation during repair^4^. *In vivo* administration of the nonsteroidal anti-inflammatory drug indomethacin, which inhibits the Wnt/β-catenin pathway, before CPP-induced injury led to significant decrease in KRT14^pos^ cell proliferation and, consequently, numbers (Fig. 6a). Moreover, *in vitro* assays showed significantly reduced clonogenic capacity (Fig. 6b), while Wnt/β-catenin inhibitors prevented the KRT14^pos^ cell proliferation and explant tissue growth in a dose-dependent manner (Fig. 6c,d). While indomethacin is not a specific Wnt/β-catenin inhibitor, knockdown of β-catenin with small hairpin RNAs (shRNAs) confirmed these observations (Fig. 6e,f).

### KRT14^pos^ cells are cells of origin of bladder cancer

**Figure 7:**
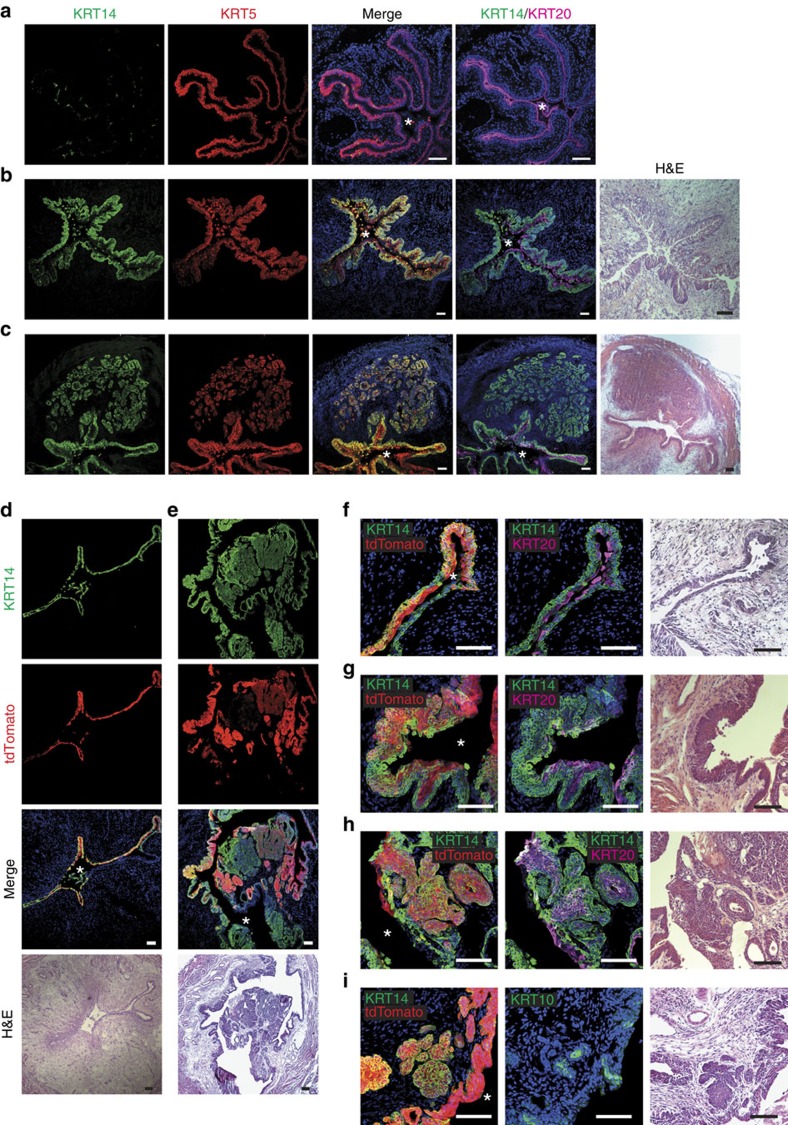
KRT14^pos^ cells are the cells of origin of bladder cancer. (**a**) IF on bladders from untreated control 8-month-old mouse. (**b**) Widespread carcinoma *in situ* (CIS) in the bladder of a 6-month-old mouse treated with BBN for 4 months, showing remarkable KRT14^pos^ cell population expansion, and extensive KRT14 and KRT5 colocalisation. (**c**) Invasive urothelial carcinoma (UC) in the bladder of an 8-month-old mouse treated with BBN for 6 months, showing extensive colocalisation of KRT14 and KRT5. (**d**-**i**) Krt14^CreERT2/+^;R26^tdTomato/+^ mice treated with tamoxifen before BBN treatment for 4 (**d**,**f**) or 6 months (**e**,**g**-**i**) showing variable histological abnormalities and cytokeratin profile. Stars designate the bladder lumen. Scale bars, 100 μm.

Previous reports have implicated KRT5^pos^ and SHH^pos^ cells as cells of origin in bladder cancer^14^,^15^,^18^,^19^. To investigate the role of KRT14^pos^ cells in tumour initiation, we employed the well-established model of chemical carcinogenesis with *N*-butyl-*N*-(4-hydroxybutyl)nitrosamine (BBN). A cohort (*n*=11) of male and female littermates (*Krt14*^CreERT2/+^, *Krt14*^CreERT2/+^;*R26*^tdTomato/+^ and *R26*^tdTomato/+^) were exposed to BBN for a maximum of 6 months. In comparison to age-matched control mice, a marked increase of KRT14^pos^ cell numbers was observed in mice exposed to the carcinogen for 4 months (Fig. 7a,b). After 6 months on BBN, animals developed invasive tumours that almost exclusively expressed Krt5 as previously described^15^. Krt5 expression absolutely coincided with Krt14 expression (Fig. 7c; Supplementary Fig. 4). We consider Krt14 positivity an indication of the cell of origin rather than an acquired property. It is important in this aspect to emphasize that occasional squamous metaplasia marked by Krt10 expression was also observed (Supplementary Fig. 4). In support of this hypothesis, lineage-tracing experiments in *Krt14*^CreERT2/+^;*R26*^tdTomato/+^ mice (*n*=7) that were injected with tamoxifen before BBN exposure showed that initial expansion of Tomato^pos^ cells (Fig. 7d) was followed by the development of neoplasms half of which (12/24) showed Tomato fluorescence (Fig. 7e–i). We observed no significant difference in regard to contribution of KRT14^pos^ cells to different tumour subtypes (Supplementary Table 4). Because cohort mice were injected with tamoxifen before BBN exposure, Tomato positivity is enough proof that Tomato^pos^ tumours originated from KRT14^pos^ cells. However, clonality is difficult to establish in this experimental set-up, and therefore, additional experiments will be required to assess the contribution of individual bladder populations in chemical-induced tumorigenesis.

## Discussion

It has been postulated for years that basal cells are responsible for the regeneration of all urinary bladder layers, including the umbrella layer^4^,^20^,^21^. Recent reports, however, favour an alternative hypothesis that the umbrella layer is regenerated by its own progenitor pool residing in the intermediate layer, while basal cells support exclusively the basal layer^5^,^14^. Our lineage-tracing experiments indicate that descendants of KRT14^pos^ basal cells are found in the intermediate and umbrella layers, following natural cycling and injury-induced regeneration. The mere existence of Tomato^pos^ cells in the intermediate and umbrella layers is sufficient proof that basally located cells can regenerate all three layers. The fact that Tomato^pos^ cells form ‘columns' that begin in the basal layer and extend upwards to include the other two layers supports the hypothesis that regeneration traces back to basal KRT14^pos^ that are able to regenerate themselves, as well as other cells types. Moreover, our data indicate that all three layers are regenerated preferentially from KRT14^pos^ cells. This is derived by the fact that while only a small fraction of basal cells is Tomato-labelled (3.89±1.25%) in the steady state, their descendants are enriched within all three layers (24.87±4.5%, 15.74±1.98% and 20.01±4.03% for the basal, intermediate and umbrella layers, respectively) after five challenges with CPP. Given the fact that not all KRT14^pos^ cells are genetically labelled with Tomato, these data rather underestimate the contribution of KRT14^pos^ cells in bladder regeneration. The apparent contradiction between our findings and published research is not straightforward to explain. A possible explanation could be that different genetic tools for conditional cell labelling show variable degree of recombination and consequently labelling efficiency.

Because our results do not eliminate the possibility that stem cells dedicated to umbrella layer regeneration actually exist, we hypothesize that these cells, if existent, can only be short-term urothelial stem cells (USCs) sufficient to regenerate mildly injured bladders, or even undisturbed aging bladders. KRT14^pos^ cells, on the other hand, represent USCs with long-term repopulating capacity (urothelial stem cells) with clear roles in tissue repair, as well as tumorigenesis. This stem cell pool is mobilized under conditions of repeated and/or chronic injury and fully regenerates the bladder urothelium. While *in vivo* clonogenic assays through orthotopic transplantation would be required to prove that a single cell can generate a fully functional bladder, all our findings indicate that KRT14^pos^ cells likely have that capacity.

In agreement with the hypothesis that stem cells could represent cells of origin of cancer, this subpopulation expands in size in response to chemical carcinogens and undergoes neoplastic transformation that leads to the development of invasive cancer. In this respect, we believe that future studies should focus on the validation of KRT14^pos^ cells as tools in regenerative medicine and targets in cancer intervention.

## Methods

### Mice

**N**ewly developed *Krt14*^*tm(CreERT2)*^ (*Krt14*^CreERT2^) heterozygotes (see below) were crossed to *Gt(ROSA)26Sor*^*tm9(CAG-tdTomato)Hze*^ (*R26*^tdTomato^) and to *Gt(ROSA)26Sor*^*tm1(HBRGF)Awai*^ (*R26*^DTR^) to produce doubly heterozygous *Krt14*^CreERT2/+^*;R26*^tdTomato/+^ or *Krt14*^CreERT2/+^*;R26*^DTR/+^, respectively. *Krt14*^CreERT2/+^ littermates were used as controls for *in vitro* cell ablation experiments. Heterozygous *Tg(Krt5-Cre/ERT2)2lpc* (*Krt5*^CreERT2^) mice where crossed to *R26*^tdTomato/+^ to produce doubly heterozygous *Krt5*^CreERT2/+^*;R26*^tdTomato/+^. Male mice between 6 and 9 weeks of age were used for all experiments, except in BBN carcinogenesis experiments where a mixed cohort was used. Wild-type mice were in all cases of C57Bl/6 background. Animals were housed in individually ventilated cages under specific pathogen-free conditions in full compliance with FELASA (Federation of Laboratory Animal Science Associations) recommendations in the Animal House Facility of the Biomedical Research Foundation of the Academy of Athens (BRFAA, Greece). All procedures for the care and treatment of the animals were approved by the Institutional Committee on Ethics of Animal Experiments and the Greek Ministry of Agriculture.

### Generation of *Krt14-CreERT2* mice

*Krt14* homologous arms were PCR-amplified from mouse 129/Sv genomic DNA as template. The 5′ arm (3.8 kb) including the KRT14 ATG, which was precisely fused into the CreERT2 ATG, and the 3′ arm (4.4 kb) flanked a CreERT2-loxP-Neo^R^-loxP cassette in a pBluescriptSK+ backbone. The targeting construct was linearized and electroporated into 129/Sv W4 embryonic stem (ES) cells that were selected with G418 (150 μg ml^−1^) for 8 days. A total of 288 clones were picked and analysed by Southern blot for homologous recombination. Positive clones were injected into C57BL/6 blastocysts. Male chimeras were crossed to C57BL/6 females and offspring was genotyped to assess germline transmission.

### Urothelial injury

Chemical injury of the urothelium was induced by intraperitoneal injection of a CPP (Sigma) solution in phosphate-buffered saline (PBS; 250 mg kg^−1^). Bladders were collected at the indicated time points after administrating CPP. In the case of multiple rounds of injury, mice were left to recover for 14 days before CPP was re-administered.

### Lineage-tracing studies

Eight-week-old *Krt14*^CreERT2/+^;*R26*^tdTomato/+^ or *Krt5*^CreERT2/+^*;R26*^tdTomato/+^ were injected intraperitoneally with 3 mg tamoxifen (Sigma) daily, for 5 consecutive days. Labelling of KRT4^pos^ or KRT5^pos^ cells without injury was assessed 72 h after the last tamoxifen injection. For lineage tracing of labelled cells post injury, the injurious chemical was administered at least 72 h after the last tamoxifen injection. For embryonic labelling of KRT4^pos^ cells, 1 mg of tamoxifen was injected once intraperitoneally to pregnant mothers at gestation day 16.5.

### Urothelial tissue explant culture

The procedure has been previously described^22^. In brief, bladders were collected, rinsed in PBS and cut sagittally. The two halves were further cut into ∼3 mm^2^ pieces and the urothelium separated from the muscle layer carefully using forceps. Tissue fragments were spread onto 12 mm diameter, 0.4 μm pore size, polyethylene terephthalate (PET) transwell filters (Corning) with the luminal side up and the lamina propria in contact with the filter. A total volume of 0.6 ml of a 1:1 mixture of MDCB153 and advanced Dulbecco's modified essential medium (Sigma), supplemented with 0.1 mM ethanolamine (Sigma), 0.1 mM phosphoethanolamine (Sigma), 0.5 μg ml^−1^ hydrocortisone (Sigma), 5 μg ml^−1^ insulin (Sigma), 15 μg ml^−1^ adenine (Sigma), 100 U ml^−1^ penicillin and 100 μg ml^−1^ streptomycin, was added to the lower compartment so that the medium was just in contact with the porous membrane, and explants were grown on the air–liquid interface. Media were changed every other day. For *ex vivo* CreERT2 activation, 4OHT (Sigma) was supplemented to the medium at 0.5 μM and medium was exchanged with 4OHT-free medium after 12 h. For KRT14^pos^ cell ablation experiments, DT (Sigma) was supplemented to the medium at 50 ng ml^−1^ and fresh DT-supplemented medium was changed daily. For the effect of the Wnt/β-catenin signalling inhibition in explant cultures, indomethacin was added to the cultures at the indicated concentrations and medium was changed every other day.

### Matrigel culture

Bladders were dissected, cutting off the ureters and the urethra just below the bladder neck. They were everted through the neck of the bladder using dissection forceps and rinsed in PBS. Everted bladders were placed in 5 mg ml^−1^ dispase II (Sigma) in growth medium and incubated at 37 °C for 1 h. Urothelial cells were collected by gently scraping with a scalpel blade, and the muscle and lamina propria layers were discarded. Urothelial cell sheets were further dissociated by pipetting up and down for 5 min and rinsed twice in PBS. Cells were either sorted directly into growth medium (see below), using a Becton Dickinson FACS Aria IIu cell sorter and collected by centrifugation, or directly suspended in 1 mM ethylenediaminetetraacetic acid (EDTA) in PBS at a density of 1,000 cells μl^−1^, appropriate volume of which was mixed with 40-μl ice-cold Matrigel (Corning) and plated onto glass coverslips in 24-well tissue culture plates. After allowing Matrigel to solidify for 20 min at 37 °C, a 1:1 mixture of MDCB153/advanced Dulbecco's modified essential medium (described above) and V79 lung fibroblast conditioned medium^4^ was added. Medium was changed every 2 days. Cells were isolated from three biological replicates consisting of two bladders each and plated at least four wells from each replicate. For the Wnt/β-catenin signalling inhibition, indomethacin was supplemented to the medium at 100 μM and medium was changed every other day. Data presented are mean values±s.e.m.

### Histology and antibodies

Tissues were fixed in 4% paraformaldehyde at 4 °C for 2 h, thoroughly washed in PBS, placed in 30% sucrose overnight and frozen in optimal cutting temperature (OCT) compound (Tissue Tek, Sakura). Frozen 10 μm sections were obtained using a Leica (CM1950) cryostat. Sectioned tissues were fixed for 5 min in 4% paraformaldehyde, washed three times in PBS and blocked in 1% bovine serum albumin, 0.1% Triton X-100 in PBS (PBT) for 1 h. Primary antibodies were added at the appropriate dilutions in PBT and tissues were incubated overnight at 4 °C in humidified chambers. Primary antibodies were washed three times in PBS, 0.1% Triton X-100 (PT), and tissues incubated with secondary antibodies in PBT for 2 h at room temperature. After three washes in PT, sections were counterstained with 4,6-diamidino-2-phenylindole (Sigma) for 3 min, rinsed in PT and mounted with Vectashield (Vector Laboratories). Matrigel spheres were fixed for 15 min at −20 °C in a 1:1 methanol:acetone mixture, followed by careful rinsing twice in PBS, embedding in OCT compound and sectioning as above. All antibodies have been previously reported in the literature. Catalogue numbers and dilutions of antibodies used in this study: chicken anti-Krt14 (Biolegend #906001, 1:250), rabbit anti-Ki67 (Abcam #ab15580, 1:150), rabbit anti-Krt5 (Biolegend #905501, 1:1000), guinea pig anti-Krt20 (Progen Biotechnik #GP-K20, 1:500) and guinea pig anti-Krt10 (Progen Biotechnik #GP-K10, 1:200). All secondary antibodies (Jackson Immunoresearch) were diluted 1:500. Hematoxylin–eosin stains were performed using standard histology procedures.

### Quantitative PCR with reverse transcription

Tissues were frozen in liquid nitrogen, pulverized with a mortar and pestle and total RNA was isolated using Nucleospin RNA (Macherey-Nagel). In the case of Matrigel-grown spheres, Matrigel was first digested using dispase II (5 mg ml^−1^) and spheres collected by centrifugation. Complementary DNA samples were prepared using Superscript II (Invitrogen), and quantitative PCR reactions were performed using KAPA SYBR FAST qPCR Master Mix (Kapa Biosystems) in a Roche LightCycler 96. Three independent biological samples were quantified in technical duplicates and expression values were normalized to glyceraldehyde 3-phosphate dehydrogenase (Gapdh). The following quantitative PCR oligos were used:

Ctnnb1_F: 5′-TGG CCT CTG ATA AAG GCA AC-3′

Ctnnb1_R: 5′-GCC CTC CAC AAA CTG CTG-3′

Axin2_F: 5′-ATG CAA AAG CCA CCC AAA GG-3′

Axin2_R: 5′-TGC ATT CCG TTT TGG CAA GG-3′

Gapdh_ F: 5′-CTG CCC AGA ACA TCA TCC CT-3′

Gapdh_ R: 5′-ACT TGG CAG GTT TCT CCA GG-3′

### Pharmacological treatment

Indomethacin (Santa Cruz) was administered by intraperitoneal injection at 2.5 mg kg^−1^ every 12 h. Mice were given four doses of indomethacin before CPP administration. Dosing scheme described above was continued after CPP administration, until mice were sacrificed at indicated time points. DT (0.04 mg kg^−1^) was injected intraperitoneally for two consecutive days and mice killed 24 h later. CPP administration to DT-treated mice was at 24 h after initial DT treatment.

### shRNA knockdown of β-catenin

shRNA sequence (CTAACCTCACTTGCAATAATccatggATTATTGCAAGTGAGGTTAG) was cloned into pLKO.1/IRESegfp. Lentiviral supernatants were generated in HEK293T cells using standard procedures and concentrated using Amicon Ultra-15 centrifugal filters (Millipore). Using serially diluted lentiviral samples and primary mouse bladder cells cultured in Matrigel, we titrated our viral preparations based on abundance of green fluorescent protein expression. Equivalent titres of shRNA and scrambled sequence-expressing particles were then used to transduce primary mouse bladder cells. In short, cells were incubated with the viral particles at 37 °C for 1 h, then mixed with Matrigel and plated. Lentiviral particles were maintained in the medium for 24 h before being removed and medium replaced. Seventy-two hours after initial transduction, all cells expressed green fluorescent protein. To eliminate any untransduced background, cells were selected using 1.5 μg ml^−1^ puromycin for 5 days.

### BBN carcinogenesis

BBN was diluted to 0.05% and administered to mice through drinking water. Mice were put on BBN water at least 72 h after the last tamoxifen injection. As separate tumours, we considered lesions within the same bladder if they were separated by normal-looking urothelium, or were uniformly positive or negative for Tomato (where relevant), or were of distinctive histology and/or marker expression pattern. All tumours with at least 50% Tomato^pos^ cells were counted as Tomato^pos^.

### Imaging

All images were captured on Leica TCS SP5 inverted confocal, Leica HC, Leica DM IRE2, Leica DM LS2 or Nikon SM2800. Image processing and cell counts were performed using ImageJ and Adobe Photoshop CS3.

### Statistical analysis

Animals were randomly assigned into different groups. Group allocation and outcome assessment was not blinded. All quantitation was performed on at least three independent biological samples, using the ImageJ software. For quantitation of urospheres in Matrigel, a lower cutoff of 0.002 mm^2^ of sphere surface was used. Data presented are mean values±s.e.m. Statistical analysis was performed using the GraphPad Prism software v6. In two group comparisons, statistical significance was determined using a two-tailed Student's *t*-test, considering a value of *P*<0.05 as significant. Multiple comparisons were performed using the Kruskal–Wallis statistical test. All sample sizes met the minimum requirements of the respective statistical test used.

### Data availability

Data supporting the findings of this study are available within the article and its supplementary information files and from the corresponding author upon reasonable request.

## Additional information

**How to cite this article:** Papafotiou, G. *et al.* KRT14 marks a subpopulation of bladder basal cells with pivotal role in regeneration and tumorigenesis. *Nat. Commun.* 7:11914 doi: 10.1038/ncomms11914 (2016).

## Supplementary Material

Supplementary Information Supplementary Figures 1-4 and Supplementary Tables 1-4

## Figures and Tables

**Table 1:** KRT14^pos^ cell counts during CPP-induced injury and repair (Fig. 1d).

	**% KRT14**^**pos**^ **cells**±**s.e.m.**	**Total cells counted**	***P*** **value**
N/T (*n*=6)	4.07±1.28	1,842	
6 h (*n*=8)	6.15±1.18	3,020	*P*>0.05
18 h (*n*=5)	13.26±1.45	1,349	*P*<0.005
24 h (*n*=6)	20±2.25	1,421	*P*<0.0005
48 h (*n*=6)	22.34±2.17	1,910	*P*<0.0001
120 h (*n*=5)	20.70±2.56	1,593	*P*<0.0005
240 h (*n*=4)	8.75±2.93	746	*P*>0.05

N/T, not treated.

**Table 2:** tdTomato^pos^ cell counts in untreated mice and after one or five rounds of CPP-induced injury and repair in the basal, intermediate and superficial cell populations in Krt14^CreERT2/+^;R26^tdTomato/+^ mice (Fig. 2d).

**Basal**	**% Tom**^**pos**^ **KRT5**^**pos**^**/total KRT5**^**pos**^±**s.e.m.**	**Total KRT5**^**pos**^ **cells**	***P*** value*
N/T (*n*=3)	3.89±1.25	2,469	
1 × CPP (*n*=4)	17.33±3.07	2,376	*P*<0.05
5 × CPP (*n*=4)	24.87±4.5	2,999	*P*>0.05

**Intermediate**	**% Tom**^**pos**^**KRT5**^**neg**^ **KRT20**^**neg**^**/total KRT5**^**neg**^ **KRT20**^**neg**^±**s.e.m.**	**Total KRT5**^**neg**^ **KRT20**^**neg**^ **cells**	***P*** **value**
N/T (*n*=3)	0	345	
1 × CPP (*n*=4)	4.58±2.17	144	*P*>0.05
5 × CPP (*n*=4)	15.74±1.98	269	*P*<0.05

**Superficial**	**% Tom**^**pos**^**KRT20**^**pos**^**/total KRT20**^**pos**^±**s.e.m**	**Total KRT20**^**pos**^ **cells**	***P*** **value**
N/T (*n*=3)	0	357	
1 × CPP (*n*=4)	5.54±0.96	492	*P*<0.01
5 × CPP (*n*=4)	20.01±4.03	512	*P*<0.05

^*^P values are calculated from comparison of 1 × and 5 × CPP treatment with steady-state untreated (N/T) cell counts.
