# Supplementary material for: KRT14 marks a subpopulation of bladder basal cells with pivotal role in regeneration and tumorigenesis
Source: Nat Commun. 2016 Jun 20;7:11914. doi: 10.1038/ncomms11914 (PMC4915139; doi:10.1038/ncomms11914)
Supplement: Supplementary Information — Supplementary Figures 1-4 and Supplementary Tables 1-4 [file ncomms11914-s1.pdf]

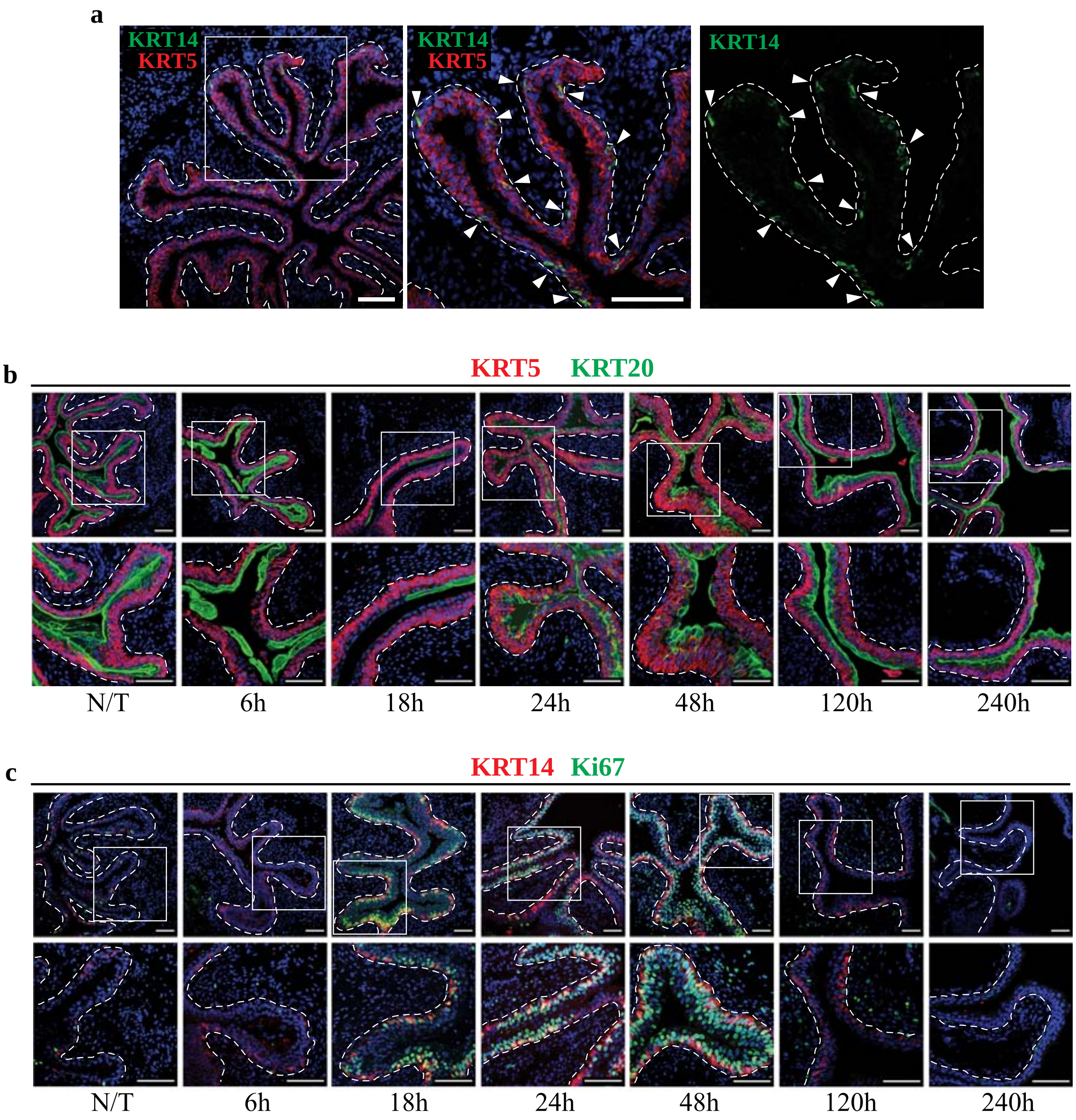

Supplementary Figure 1| Dynamic changes of bladder subpopulations during CPP-induced injury and repair. (**a**) IF against KRT14 and KRT5 in an 8 week-old wild type mouse. (**b**) IF against KRT5 and KRT20 at distinct time points during damage and repair at 20x and 40x magnifications. (**c**) IF against KRT14 and Ki67 at the above time points. Images for each time point in **a,** and **b,** come from adjacent 10 μm sections and show equivalent regions wherever available. Dashed lines represent the basement membrane. Scale bars represent 100 μm.

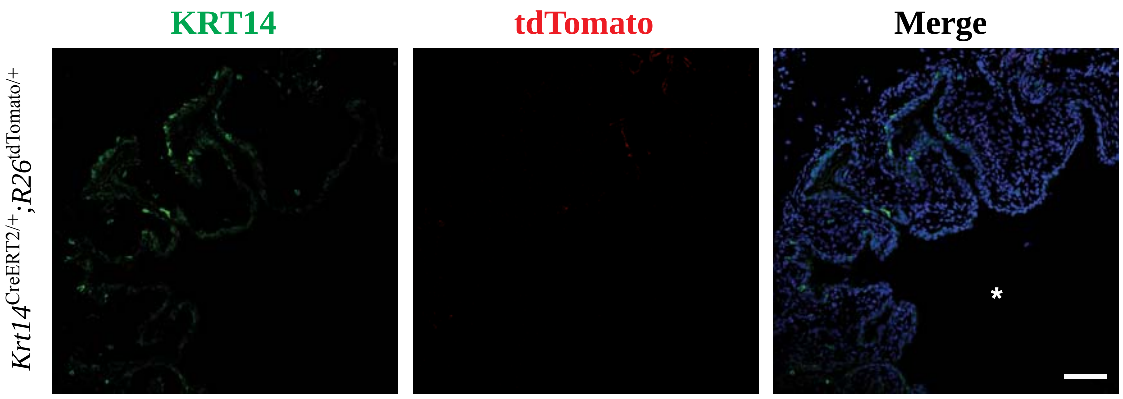

Supplementary Figure 2 | Genetic labeling of basal subtypes in mouse bladders. Section of a bladder from a *Krt14*$^{CreERT2/+}$;*R26*$^{tdTomato/+}$ mouse without Tamoxifen activation, showing lack of tdTomato expression. Stars designate the bladder lumen. Scale bar represents 100 μm.

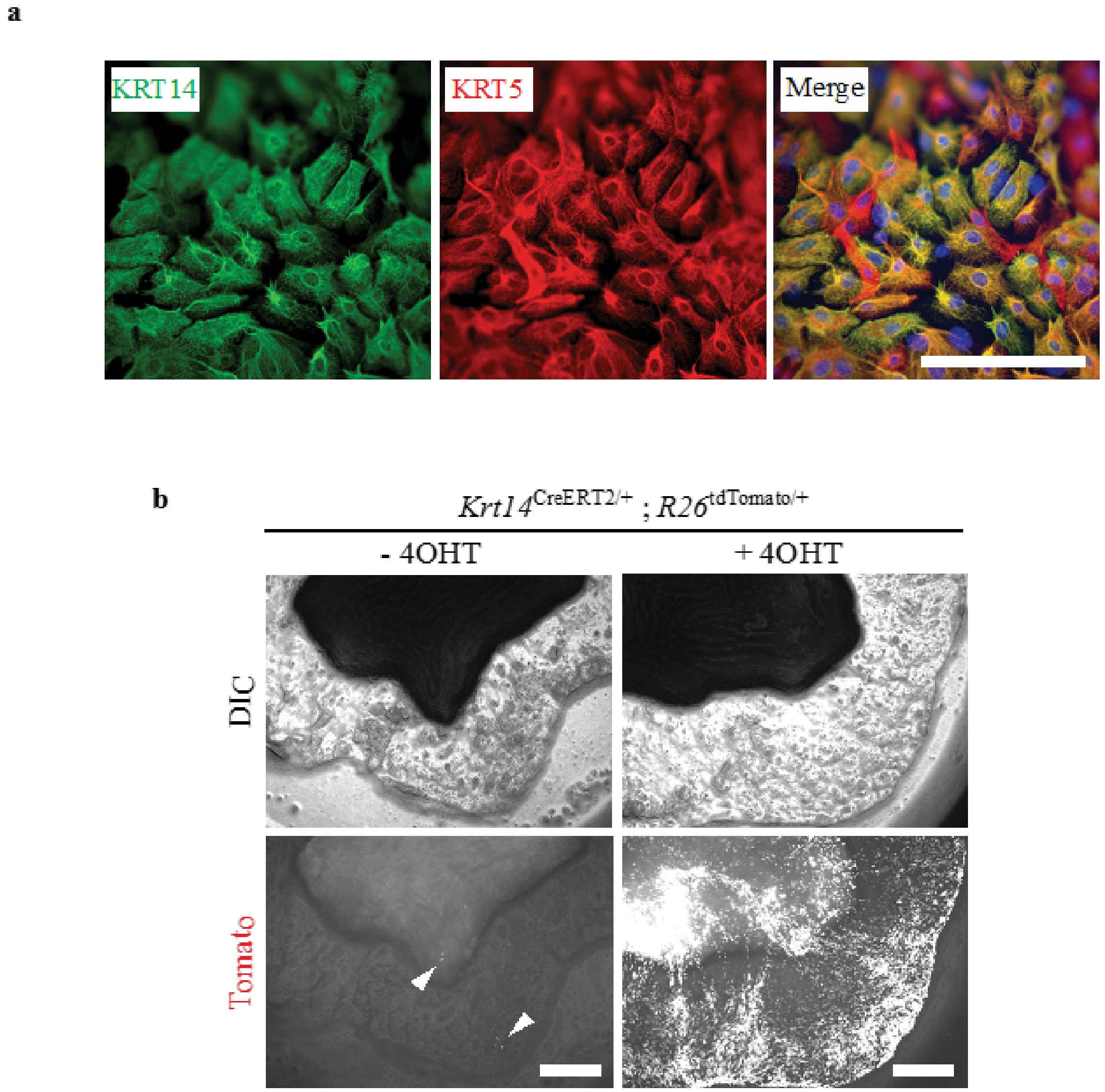

Supplementary Figure 3 | KRT14^{pos} cells support bladder explant growth ex vivo.
(**a**) Tamoxifen administration either *in vivo* or *ex vivo* is necessary for CreERT2 transgene activation and subsequent tomato expression. Arrowheads point to extremely sparse escapers. (**b**) Outgrowth cells are KRT14^{pos} and KRT5^{pos}. Scale bars represent 1 mm in **a,** and 100 µm in **b**.

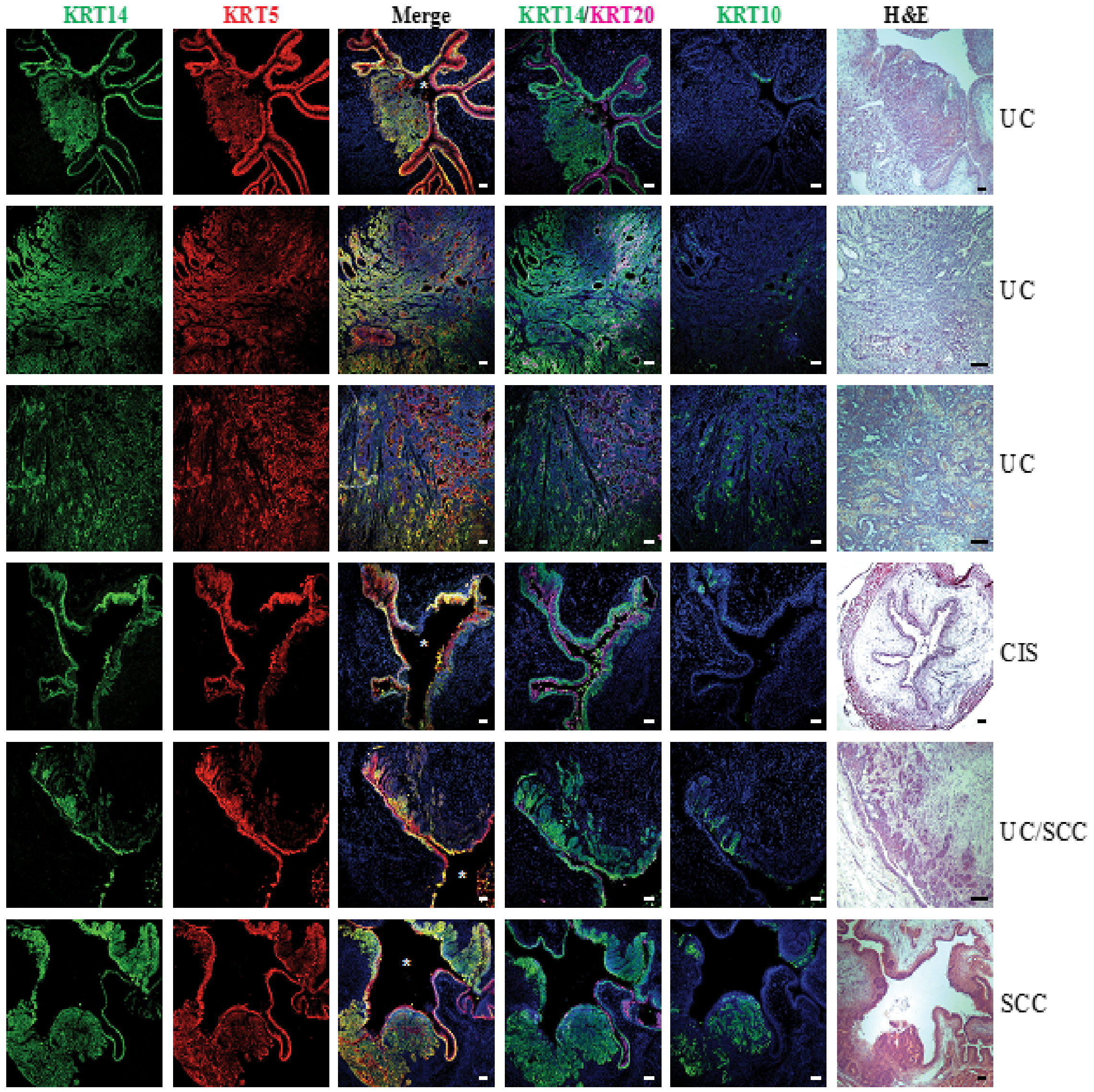

Supplementary Figure 4 | BBN-induced bladder tumors express KRT14.
BBN treatment induces development of different stage neoplasms. The majority co-express KRT5 and 14, while occasional KRT20 and KRT10 positivity is observed. UC: urothelial carcinoma; CIS: carcinoma in situ; SCC: Squamous cell carcinoma. Stars designate the bladder lumen. Scale bars represent 100 µm.

Supplementary Table 1. Ki67^{pos} cell counts during CPP-induced injury and repair in total epithelial cells (Fig.1e)

	**% total epithelial proliferation ± s.e.m.**	**Total cells counted**	***P* value**
**N/T** (n=5)	2.14 ± 0.92	1563	
**6h** (n=4)	0.49 ± 0.31	1879	$P > 0.05$
**18h** (n=3)	31.21 ± 7.26	839	$P < 0.005$
**24h** (n=6)	36.55 ± 7.03	1342	$P < 0.005$
**48h** (n=6)	66.26 ± 7.94	1532	$P < 0.001$
**120h** (n=6)	2.99 ± 0.81	1951	$P > 0.05$
**240h** (n=3)	1.33 ± 0.35	463	$P > 0.05$

Supplementary Table 2. Ki67^{pos} cell counts during CPP-induced injury and repair in the KRT14^{pos} and KRT14^{neg} subpopulations (Fig.1f)

	**% KRT14^{pos} proliferation ±s.e.m.**	**KRT14^{pos} cells**	**% KRT14^{neg} proliferation ±s.e.m.**	**KRT14^{neg} cells**	***P* value**
**N/T** (n=5)	11.67 ± 7.26	34	1.91 ± 0.91	1529	$P > 0.05$
**6h** (n=4)	3.02 ± 1.07	112	0.32 ± 0.32	1767	$P > 0.05$
**18h** (n=3)	75.16 ± 8.27	124	24.27 ± 7.19	715	$P < 0.01$
**24h** (n=6)	71.25 ± 9.07	230	28.87 ± 6.78	1112	$P < 0.005$
**48h** (n=6)	85.7 ± 7.84	371	60.41 ± 8.21	1161	$P < 0.05$
**120h** (n=6)	10.15 ± 4.32	328	1.34 ± 0.46	1623	$P > 0.05$
**240h** (n=3)	3.04 ± 1.52	65	0.95 ± 0.51	398	$P > 0.05$

Supplementary Table 3. tdTomato^{pos} cell counts in untreated mice and after one round of CPP-induced injury and repair in the basal, intermediate and superficial cell populations in *Krt5*$^{CreERT2/+}$*;R26*$^{tdTomato/+}$ mice (Fig. 3b)

**Basal**	**% Tom^{pos} KRT5^{pos} / total KRT5^{pos} ± s.e.m.**	**Total KRT5^{pos} cells**	***P* value**
**N/T** (n=4)	63.54 ± 8.28	3887	
**1 x CPP** (n=4)	79.74 ± 4.92	3046	$P > 0.05$
**Intermediate**	**% Tom^{pos}KRT5^{neg} KRT20^{neg} / total KRT5^{neg} KRT20^{neg} ± s.e.m.**	**Total KRT5^{neg} KRT20^{neg} cells**	***P* value**
**N/T** (n=4)	11.16 ± 5.2	853	
**1 x CPP** (n=4)	50.84 ± 5.67	783	$P < 0.01$
**Superficial**	**% Tom^{pos}KRT20^{pos} / total KRT20^{pos} ± s.e.m.**	**Total KRT20^{pos} cells**	***P* value**
**N/T** (n=4)	2.24 ± 1.11	877	
**1 x CPP** (n=4)	27.4 ± 5.32	525	$P < 0.01$

Supplementary Table 4. Lineage tracing of KRT14^{pos} cells in BBN-induced tumours

**BBN**	**4 months**		**6 months**	
**# mice**	2		5	
**tdTomato**	+	-	+	-
**Papillary**	0	0	2	0
**Hyp/Dysp**[1]	1	2	3	3
**CIN/UC**[2]	1	0	3	5
**SCC**[3]	0	0	2	2

[1]Hyperplasia/Dysplasia
[2]Carcinoma In Situ/Urothelial Carcinoma
[3]Squamous Cell Carcinoma
